# Supplementary material for: Novel effects of Ras-MAPK pathogenic variants on the developing human brain and their link to gene expression and inhibition abilities
Source: Transl Psychiatry. 2023 Jul 6;13:245. doi: 10.1038/s41398-023-02504-4 (PMC10322993; doi:10.1038/s41398-023-02504-4)
Supplement: Supplementary file 1 — Supplemental Material [file 41398_2023_2504_MOESM1_ESM.docx]

**Supplementary Material**

**Methods and Materials**

**Participants**

Identical magnetic resonance imaging scanning and behavioral assessment protocols were used in both studies. All NS participants were required to submit prior medical documentation and genetic testing results for either *PTPN11* mutation or *SOS1* mutation to qualify for study inclusion. Participants with NS were recruited primarily through the Noonan Syndrome Foundation and Noonan Syndrome social media groups, in addition to a small number referred from physicians in relevant specialties nationwide. TD controls were recruited from a longitudinal Turner Syndrome study also at Stanford University School of Medicine via parent organizations and local flyer advertisements. For the *PTPN11* group, the sample size (*n*=30) was determined by a power analysis of data from our preliminary study of children with NS (*n*=12). The *SOS1* analysis is preliminary in nature and therefore, has a sample size limitation (*n*=10). Despite its limited sample size, the inclusion of the SOS1 group in our cohort presents an unprecedented opportunity to study how two distinct genotypes of NS alter brain anatomy. Potential participants for both the NS and TD groups were excluded due to premature birth (gestational age under 32 weeks), low birth weight (less than 2000 g), known major psychiatric disorder diagnosis, and/or any MRI contraindications. Other exclusion criteria included a history of neurological disorders known to impact cognitive development or brain structure, including seizures, or a diagnosis of gross structural malformations, such as Arnold-Chiari malformations. 17 children with NS were excluded based on these criteria and 6 children with NS were excluded due to age prior to participating in the study.

**Medication History and Tanner Staging**

Physical characteristics of NS include short stature, facial abnormalities, and cardiac defects. Thus, medication status of each participant was recorded; 13 NS participants were taking growth hormone, 6 NS participants were taking stimulants, and 3 NS participants were taking SSRIs for the duration of the study (Table 1). Participants’ pubertal developmental statuses using Tanner stages^1^ was assessed by an experienced physician (TG). For both TD and NS groups, only participants at Tanner stages of 2 and below were included in the study to control for the effect of pubertal status on brain anatomy (Table 1).

**Cognitive and Behavioral Assessment**

The neurodevelopmental, disruptive, and conduct disorders supplement of the Kiddie Schedule for Affective Disorders and Schizophrenia Present and Lifetime (K-SADS-PL) was administered by a trained clinician (TG) to all participants in the NS group to obtain psychiatric diagnoses^2^.

**MRI**

T1-weighted structural scans were repeated until a high-quality image (i.e., minimal to no motion and no wrap around or other artifacts) was acquired. Trained raters manually inspected scans for image quality and selected the highest quality image for morphometric analysis with FreeSurfer. To quantitatively assess initial image quality, we computed cumulative Euler numbers (sum of Euler numbers for left and right hemispheres) for all subjects. We then performed a student’s *t*-test between our NS (*n*=40) and TD (*n*=40) groups after excluding subjects with left or right hemispheric Euler numbers above a threshold of -217^3^.

**Table S1.** **BASC-2 Primary Subscales**

|  | ***PTPN11*** | ***SOS1*** | **Typically Developing** | ***p* value**^a^ | ***p* value**^b^ |
| --- | --- | --- | --- | --- | --- |
| Attention Problems^c^ | 59.79±10.38 | 62.50±11.93 | 51.68±10.28 | *p* < 0.01 | *p* < 0.05 |
| Hyperactivity^c^ | 63.28±14.07 | 63.20±16.43 | 48.76±11.93 | *p* < 0.001 | *p* < 0.05 |
| Anxiety^c^ | 56.24±10.92 | 57.60±19.02 | 51.87±12.22 | ns | ns |
| Depression^c^ | 59.17±11.99 | 57.00±21.69 | 50.11±12.51 | *p* < 0.01 | ns |
| Somatization^c^ | 59.55±12.81 | 71.80±15.19 | 48.87±13.22 | *p* < 0.01 | *p* < 0.001 |
| Atypicality^c^ | 60.45±15.16 | 62.30±17.37 | 49.45±11.75 | *p* < 0.01 | *p* < 0.05 |
| Withdrawal^c^ | 58.21±9.75 | 57.00±14.55 | 51.71±13.18 | *p* < 0.05 | ns |
| Adaptability^c^ | 40.97±9.87 | 43.00±12.75 | 48.53±11.01 | *p* < 0.01 | ns |
| Social Skills^c^ | 46.21±9.43 | 49.50±15.67 | 50.18±10.11 | ns | ns |
| Leadership^d^ | 41.33±8.27 | 46.78±12.62 | 53.85±7.35 | *p* < 0.001 | ns |
| Activites of Daily Living^c^ | 36.86±10.06 | 39.10±14.36 | 49.03±10.83 | *p* < 0.001 | ns |
| Functional Communication^c^ | 41.10±9.73 | 44.20±13.30 | 50.39±8.53 | *p* < 0.001 | ns |
| Aggression^c^ | 56.10±10.63 | 53.30±12.14 | 50.21±9.37 | *p* < 0.05 | ns |
| Conduct Disorder^d^ | 55.29±13.35 | 55.56±11.04 | 49.59±9.25 | ns | ns |

All values are reported in mean ± standard deviation; Welch’s two-sample *t*-test was used to assess significance between groups; ns, not significant.

^a^Children with *PTPN11* compared with controls

^b^Children with *SOS1* compared with controls

^c^*n* of 29 for *PTPN11*, *n* of 10 for *SOS1*, and *n* of 38 for TD

^d^*n* of 24 for *PTPN11*, *n* of 9 for *SOS1*, and *n* of 34 for TD, Leadership and Conduct Disorder not administered to children age <5

**Table S2**. ***P* values for Parcellated Brain Regions**

|  | LEFT | | | RIGHT | | | |
| --- | --- | --- | --- | --- | --- | --- | --- |
| Brain Region^a^ | **GMV** | **SA** | **CT** | | **GMV** | **SA** | **CT** |
| *SUBCORTICAL* |  |  |  | |  |  |  |
| Caudate | **-0.00602** | - | - | | **-0.00012**** | - | - |
| Putamen | **-0.00031**** | - | - | | **-0.00012**** | - | - |
| Pallidum | **-0.00012**** | - | - | | **-0.00015**** | - | - |
| Hippocampus | -0.79194 | - | - | | **-0.02916** | - | - |
| Amygdala | -0.60187 | - | - | | 0.90260 | - | - |
| *FRONTAL* |  |  |  | |  |  |  |
| Caudal middle frontal | **-0.00047**** | **-0.01182** | -0.11565 | | -0.51882 | -0.99370 | -0.21793 |
| Lateral orbitofrontal | 0.99897 | 0.50971 | 0.28261 | | -0.95333 | -0.74035 | 0.34096 |
| Medial orbitofrontal | 0.96041 | -0.62319 | 0.06239 | | -0.96722 | -0.64752 | -0.06239 |
| Paracentral | 0.66557 | 0.12037 | -0.44713 | | -0.92115 | 0.36998 | -0.30745 |
| Parsopercularis | -0.09166 | -0.45755 | **-0.00488*** | | -0.95333 | 0.74035 | -0.64088 |
| Parsorbitalis | -0.99897 | 0.46471 | 0.52295 | | -0.96041 | -0.84643 | **0.00793** |
| Parstriangularis | -0.96722 | 0.46421 | -0.73455 | | -0.49036 | -0.46471 | 0.72208 |
| Precentral | **-0.01195** | -0.46471 | **-0.04876** | | **-0.01594** | -0.55461 | **-0.01623** |
| Rostral middle frontal | -0.44677 | 0.99370 | -0.95153 | | -0.82899 | -0.46471 | **0.00793** |
| Superior frontal | -0.75985 | 0.45755 | 0.84248 | | 0.37550 | **0.00835** | 0.93657 |
| Frontal pole | -0.15605 | 0.99370 | 0.61917 | | -0.17102 | -0.46471 | 0.13152 |
| *PARIETAL* |  |  |  | |  |  |  |
| Inferior parietal | 0.99897 | **0.04068** | 0.61917 | | **-0.01453** | 0.74035 | -0.10093 |
| Post central | 0.77060 | 0.07970 | -0.61060 | | -0.95333 | 0.99370 | 0.69962 |
| Precuneus | **-0.01099** | -0.05413 | 0.75312 | | -0.22182 | -0.69822 | -0.47409 |
| Superior parietal | **-0.01248** | **-0.04929** | 0.59131 | | **-0.00156*** | -0.11669 | **-0.04511** |
| Supramarginal | 0.96041 | **0.04929** | -0.88201 | | -0.75985 | 0.12037 | -0.06239 |
| *CINGULATE* |  |  |  | |  |  |  |
| Caudal anterior cingulate | 0.56132 | 0.99370 | 0.37090 | | -0.95333 | -0.81643 | 0.07729 |
| Isthmus cingulate | -0.43150 | 0.64589 | -0.37090 | | -0.86195 | 0.99370 | -0.51414 |
| Posterior cingulate | 0.95333 | 0.35118 | 0.63702 | | **-0.02325** | -0.24207 | 0.08766 |
| Rostral anterior cingulate | 0.52017 | 0.82381 | 0.24604 | | 0.96041 | -0.56447 | 0.11609 |
| *TEMPORAL* |  |  |  | |  |  |  |
| Banks STS^b^ | -0.33377 | 0.06473 | 0.89405 | | -0.96041 | 0.34679 | -0.48227 |
| Entorhinal | **-0.02187** | **-0.01182** | 0.06239 | | **-0.00118*** | **-0.00835** | 0.95153 |
| Fusiform | -0.33605 | -0.45755 | 0.43399 | | -0.07049 | -0.36068 | -0.66261 |
| Inferior temporal | -0.92115 | 0.87219 | -0.66329 | | **-0.01099** | -0.07970 | -0.18015 |
| Middle temporal | 0.92115 | **0.04701** | -0.75312 | | -0.75985 | 0.45755 | -0.95153 |
| Parahippocampal | -0.99897 | **0.00231*** | **-0.01623** | | 0.96722 | **0.04929** | **-0.00886** |
| Superior temporal | -0.90952 | 0.07287 | **-0.04876** | | 0.62745 | **0.02241** | 0.61917 |
| Temporal pole | **-0.00973** | -0.21090 | -0.51414 | | **-0.00118*** | **-0.00231*** | 0.73455 |
| Transverse temporal | 0.62745 | 0.46471 | 0.61917 | | 0.92115 | 0.89582 | 0.61917 |
| Insula | 0.96041 | -0.71343 | **0.02494** | | 0.95333 | -0.50971 | **0.01019** |
| *OCCIPITAL* |  |  |  | |  |  |  |
| Cuneus | **-0.00211*** | **-0.04365** | 0.61917 | | -0.34958 | -0.46822 | 0.75312 |
| Lateral occipital | 0.34958 | 0.40822 | **0.00488*** | | 0.77060 | 0.99370 | 0.05442 |
| Lingual | **-0.00143*** | -0.32827 | -0.28261 | | **-0.00118*** | -0.12037 | **-0.01759** |
| Pericalcarine | **-0.00325*** | -0.11424 | -0.50915 | | **-0.00156*** | **-0.04068** | -0.09502 |

^a^34 brain regions were parcellated using FreeSurfer software. Children with Noonan syndrome and *PTPN11* mutation were compared to TD controls, values represent FDR-corrected *p*-values and sign of *p*-values represents direction of differences (negative: NS < TD; positive: NS > TD); Bolded value with no asterisk has a *p*-value of <0.05, a bolded value with * has a *p*-value of <0.005, and finally, a bolded value with ** has a *p*-value of <0.001.

^b^Banks of the superior temporal sulcus

**Table S3**. **Effect sizes for Parcellated Brain Regions**

|  |  |  | | LEFT | | |  | |  | | | |  | | RIGHT | | | |  |
| --- | --- | --- | --- | --- | --- | --- | --- | --- | --- | --- | --- | --- | --- | --- | --- | --- | --- | --- | --- |
| Brain Region^a^ | **GMV** | | | | **SA** | | | **CT** | | | **GMV** | | | **SA** | | | **CT** | | |
| Group ( vs. TD) | ***PTPN11*** | | ***SOS1*** | | ***PTPN11*** | ***SOS1*** | | ***PTPN11*** | | ***SOS1*** | ***PTPN11*** | ***SOS1*** | | ***PTPN11*** | | ***SOS1*** | ***PTPN11*** | ***SOS1*** | |
| *SUBCORTICAL* |  | |  | |  |  | |  | |  |  |  | |  | |  |  |  | |
| Caudate | **-0.7044** | | -0.2695 | | - | - | | - | | - | **-1.0294**** | -0.3585 | | - | | - | - | - | |
| Putamen | **-0.8582**** | | -0.3255 | | - | - | | - | | - | **-0.9060**** | -0.8066 | | - | | - | - | - | |
| Pallidum | **-0.9019**** | | **-0.7982** | | - | - | | - | | - | **-0.8831**** | **-1.0285** | | - | | - | - | - | |
| Hippocampus | -0.1098 | | -0.0303 | | - | - | | - | | - | **-0.5345** | -0.2392 | | - | | - | - | - | |
| Amygdala | -0.1771 | | 0.0063 | | - | - | | - | | - | 0.0449 | 0.3144 | | - | | - | - | - | |
| *FRONTAL* |  | |  | |  |  | |  | |  |  |  | |  | |  |  |  | |
| Caudal middle frontal | **-1.0602**** | | -1.0584 | | **-0.8228** | **-0.9559** | | -0.4290 | | -0.0311 | -0.2594 | -0.5674 | | 0.0154 | | -0.3317 | -0.3811 | -0.1722 | |
| Lateral orbitofrontal | 0.0306 | | -0.5686 | | 0.1842 | -0.6536 | | 0.3302 | | 0.1234 | -0.0495 | -0.6239 | | -0.1334 | | -0.8553 | 0.3406 | 0.7992 | |
| Medial orbitofrontal | 0.0521 | | 0.4077 | | -0.2209 | 0.0288 | | 0.6157 | | 0.6027 | -0.0036 | 0.1033 | | -0.1841 | | -0.2497 | 0.5769 | 0.8909 | |
| Paracentral | 0.2311 | | -0.4298 | | 0.5226 | -0.0094 | | -0.2637 | | **-0.8212** | -0.0878 | -0.4319 | | 0.3347 | | -0.3031 | -0.3795 | 0.1897 | |
| Parsopercularis | -0.4871 | | 0.1890 | | -0.2962 | 0.6307 | | **-0.8746*** | | **-0.8181** | -0.1056 | -0.0238 | | 0.0655 | | 0.0169 | -0.1403 | 0.1191 | |
| Parsorbitalis | 0.0186 | | -0.0583 | | 0.2426 | 0.0693 | | 0.1454 | | 0.1180 | -0.0623 | -0.2873 | | -0.0424 | | 0.3741 | **0.6126** | -0.4329 | |
| Parstriangularis | -0.0262 | | 0.2373 | | 0.2504 | 0.4283 | | -0.0983 | | 0.2371 | -0.2640 | 0.1571 | | -0.2803 | | 0.6242 | 0.0325 | **-0.7354** | |
| Precentral | **-0.6954** | | -0.7546 | | -0.2386 | -0.4745 | | **-0.5798** | | -0.2281 | **-0.6667** | -0.7036 | | -0.1791 | | -0.2989 | **-0.7326** | -0.4687 | |
| Rostral middle frontal | -0.2574 | | -0.4651 | | 0.0340 | -0.2191 | | -0.0418 | | -0.3564 | -0.1395 | -0.1912 | | -0.2517 | | -0.2095 | **0.7471** | 0.1246 | |
| Superior frontal | -0.1615 | | -0.1628 | | 0.2601 | 0.3366 | | 0.0952 | | 0.0092 | 0.3179 | 0.0516 | | **0.8376** | | 0.7476 | 0.0162 | -0.1389 | |
| Frontal pole | -0.4479 | | -0.3334 | | -0.0131 | **0.6710** | | 0.0971 | | -0.4296 | -0.4125 | -0.0956 | | -0.2340 | | 0.5832 | 0.4305 | -0.1461 | |
| *PARIETAL* |  | |  | |  |  | |  | |  |  |  | |  | |  |  |  | |
| Inferior parietal | 0.0292 | | 0.1212 | | **0.7074** | 0.3741 | | 0.2443 | | 0.5618 | **-0.5819** | -0.5434 | | 0.1600 | | 0.2574 | -0.3783 | -0.5773 | |
| Post central | 0.1469 | | -0.0387 | | 0.5575 | **0.6273** | | -0.0845 | | -0.3775 | -0.0618 | -0.2784 | | 0.0091 | | 0.2504 | 0.2467 | -0.3899 | |
| Precuneus | **-0.6924** | | -0.1527 | | -0.6137 | -0.1502 | | 0.1395 | | 0.3946 | -0.3749 | 0.0463 | | -0.1091 | | 0.2848 | -0.3057 | 0.1659 | |
| Superior parietal | **-0.6629** | | 0.3487 | | **-0.6335** | 0.4461 | | 0.3313 | | 0.7549 | **-0.8865*** | -0.3421 | | -0.5574 | | -0.0305 | **-0.4546** | -0.0051 | |
| Supramarginal | 0.0937 | | 0.0704 | | **0.6680** | 0.7485 | | 0.0589 | | -0.2207 | -0.1937 | 0.2796 | | 0.4260 | | **0.9738** | -0.3939 | -0.4476 | |
| *CINGULATE* |  | |  | |  |  | |  | |  |  |  | |  | |  |  |  | |
| Caudal anterior cingulate | 0.2224 | | -0.4222 | | -0.0278 | -0.6657 | | 0.3271 | | 0.0295 | -0.0593 | -0.4273 | | -0.1382 | | -0.5924 | 0.5410 | 0.6441 | |
| Isthmus cingulate | -0.3000 | | 0.3505 | | 0.2121 | 0.2222 | | -0.3892 | | 0.1641 | -0.0843 | -0.1176 | | 0.0830 | | 0.3463 | -0.3305 | -0.6252 | |
| Posterior cingulate | 0.1157 | | -0.2367 | | 0.3793 | -0.3283 | | 0.1917 | | 0.3961 | **-0.5754** | -0.3216 | | -0.3881 | | -0.4582 | 0.5443 | 0.5945 | |
| Rostral anterior cingulate | 0.2067 | | -0.4288 | | 0.0252 | -0.7327 | | 0.4319 | | 0.2187 | 0.0412 | -0.5010 | | -0.2303 | | **-0.7210** | 0.4416 | 0.6149 | |
| *TEMPORAL* |  | |  | |  |  | |  | |  |  |  | |  | |  |  |  | |
| Banks STS^b^ | 0.3757 | | -0.1193 | | 0.6025 | -0.3687 | | 0.0802 | | **0.8878** | -0.0179 | 0.3606 | | 0.3864 | | 0.6368 | -0.2106 | **-0.6162** | |
| Entorhinal | **-0.6197** | | -0.1660 | | **-0.7920** | 0.0950 | | 0.4137 | | -0.0593 | **-0.9102*** | -0.5198 | | **-0.8540** | | -0.3798 | -0.0322 | 0.0449 | |
| Fusiform | -0.3717 | | -0.1333 | | -0.3109 | 0.0698 | | 0.2376 | | 0.3497 | -0.5140 | -0.7066 | | -0.3661 | | -0.3167 | -0.1457 | -0.3892 | |
| Inferior temporal | -0.1048 | | -0.1257 | | 0.0603 | -0.0247 | | -0.2038 | | 0.0891 | **-0.7028** | -0.9622 | | -0.5666 | | -0.6274 | -0.4013 | -0.7416 | |
| Middle temporal | 0.0880 | | 0.1363 | | **0.6250** | 0.0702 | | -0.1525 | | 0.7039 | -0.1330 | -0.2012 | | 0.2956 | | 0.2409 | -0.0133 | -0.3581 | |
| Parahippocampal | -0.0488 | | 0.3109 | | **0.9610*** | **1.2677**** | | **-0.7140** | | -0.2907 | 0.0443 | -0.1438 | | **0.6637** | | 0.3619 | **-0.8202** | -0.2610 | |
| Superior temporal | -0.0840 | | -0.4644 | | 0.6065 | 0.1333 | | **-0.5987** | | -0.5107 | 0.1938 | -0.0608 | | **0.7643** | | 0.7689 | 0.1775 | -0.5114 | |
| Temporal pole | **-0.7533** | | -1.1984 | | -0.4549 | -0.1158 | | -0.2444 | | **-0.9858** | **-0.9138*** | -0.3880 | | **-1.0232*** | | -0.1141 | -0.0128 | -0.5547 | |
| Transverse temporal | 0.2166 | | 0.0916 | | 0.2784 | -0.3287 | | 0.2984 | | **0.9644** | 0.0999 | 0.4168 | | 0.0382 | | 0.1332 | 0.2622 | 0.2795 | |
| Insula | 0.0627 | | -0.1147 | | -0.1772 | -0.2176 | | **0.7165** | | 0.4661 | 0.1087 | -0.1994 | | -0.2451 | | -0.3193 | **0.7850** | 0.2760 | |
| *OCCIPITAL* |  | |  | |  |  | |  | |  |  |  | |  | |  |  |  | |
| Cuneus | **-0.8014*** | | -0.2034 | | **-0.6259** | -0.3668 | | 0.1045 | | 0.6861 | -0.3081 | -0.2175 | | -0.2535 | | -0.1284 | 0.0266 | 0.3572 | |
| Lateral occipital | 0.3722 | | 0.8741 | | 0.3452 | 0.0243 | | **0.7754*** | | **1.7787**** | 0.1936 | 0.2107 | | 0.0133 | | -0.1910 | 0.5689 | **0.6864** | |
| Lingual | **-0.8717*** | | **-1.2819** | | -0.3390 | -0.7298 | | -0.3879 | | -0.2876 | **-0.8974*** | -1.1238 | | -0.4947 | | -0.6845 | **-0.7014** | **-0.8862** | |
| Pericalcarine | **-0.8214*** | | -0.9493 | | -0.4883 | **-0.7701** | | -0.3257 | | -0.7226 | **-0.8485*** | -0.8108 | | **-0.6737** | | -0.4299 | -0.5062 | **-0.9280** | |

^a^34 brain regions were parcellated using FreeSurfer software. Children with Noonan syndrome and either *PTPN11* or *SOS1* mutations were compared to TD controls, values represent Cohen’s *d* effect size; Bolded value with no asterisk has a *p*-value of <0.05, a bolded value with * has a *p*-value of <0.005, and finally, a bolded value with ** has a *p*-value of <0.001.

^b^Banks of the superior temporal sulcus

**Table S4**. ***P* values for Parcellated Brain Regions**

|  | LEFT | | | | RIGHT | | |
| --- | --- | --- | --- | --- | --- | --- | --- |
| Brain Region | **GMV** | **SA** | **CT** | **GMV** | | **SA** | **CT** |
| *SUBCORTICAL* |  |  |  |  | |  |  |
| Caudate | -0.599664166 | - | - | -0.599664166 | | - | - |
| Putamen | -0.599664166 | - | - | -0.059472217 | | - | - |
| Pallidum | **-0.03821175** | - | - | **-0.011731** | | - | - |
| Hippocampus | -0.987306358 | - | - | -0.599664166 | | - | - |
| Amygdala | 0.987306358 | - | - | 0.60120844 | | - | - |
| *FRONTAL* |  |  |  |  | |  |  |
| Caudal middle frontal | -0.100190265 | **-0.016151124** | -0.915782273 | -0.584670384 | | -0.432304231 | -0.603025175 |
| Lateral orbitofrontal | -0.619311693 | -0.082855667 | 0.730571478 | -0.348094608 | | -0.068261449 | 0.071297625 |
| Medial orbitofrontal | 0.688268611 | 0.93211076 | 0.052851839 | 0.883991973 | | -0.520687817 | 0.079021582 |
| Paracentral | -0.688268611 | -0.97742726 | **-0.031657991** | -0.619311693 | | -0.329970868 | 0.597039808 |
| Parsopercularis | 0.883991973 | 0.09390975 | **-0.030306492** | -0.946711393 | | 0.963389626 | 0.758005975 |
| Parsorbitalis | -0.916405464 | 0.874328759 | 0.778254784 | -0.824087262 | | 0.27756801 | -0.189132234 |
| Parstriangularis | 0.824087262 | 0.17150074 | 0.460138459 | 0.883991973 | | 0.154439248 | **-0.030597099** |
| Precentral | -0.307507309 | -0.175697272 | -0.438290984 | -0.307507309 | | -0.365507413 | -0.167849364 |
| Rostral middle frontal | -0.619311693 | -0.543523045 | -0.325367661 | -0.883991973 | | -0.580368056 | 0.77226236 |
| Superior frontal | -0.883991973 | 0.263600168 | 0.978752261 | 0.913986433 | | 0.068656255 | -0.604683578 |
| Frontal pole | -0.734015778 | **0.038561508** | -0.310514663 | -0.913986433 | | 0.158309234 | -0.759978157 |
| *PARIETAL* |  |  |  |  | |  |  |
| Inferior parietal | 0.883991973 | 0.200306528 | 0.200307483 | -0.584670384 | | 0.54018286 | -0.16537642 |
| Post central | -0.913986433 | **0.038073693** | -0.203291602 | -0.734015778 | | 0.427717049 | -0.280953014 |
| Precuneus | -0.883991973 | -0.724063154 | 0.218775851 | 0.913986433 | | 0.265640402 | 0.641756656 |
| Superior parietal | 0.734015778 | 0.29428308 | 0.067031721 | -0.734015778 | | -0.936793692 | -0.981196433 |
| Supramarginal | 0.913986433 | 0.063416037 | -0.56161024 | 0.883991973 | | **0.038862095** | -0.296801937 |
| *CINGULATE* |  |  |  |  | |  |  |
| Caudal anterior cingulate | -0.599504708 | -0.060672979 | 0.911707523 | -0.734015778 | | -0.13307023 | 0.230760959 |
| Isthmus cingulate | 0.812328159 | 0.513974678 | 0.566822001 | -0.883991973 | | 0.349125525 | -0.078881157 |
| Posterior cingulate | -0.812328159 | -0.385853867 | 0.223971889 | -0.812328159 | | -0.262258821 | 0.147178674 |
| Rostral anterior cingulate | -0.688268611 | -0.08629282 | 0.577719982 | -0.307507309 | | **-0.008567332** | 0.100729737 |
| *TEMPORAL* |  |  |  |  | |  |  |
| Banks STS^b^ | -0.883991973 | -0.379945695 | **0.018412874** | 0.734015778 | | 0.099691022 | **-0.019453446** |
| Entorhinal | -0.883991973 | 0.810829842 | -0.900846934 | -0.307507309 | | -0.29960097 | 0.895738822 |
| Fusiform | -0.883991973 | 0.884615157 | 0.389133777 | -0.307507309 | | -0.435801306 | -0.278645162 |
| Inferior temporal | -0.883991973 | -0.952418351 | 0.838322958 | -0.307507309 | | -0.141168153 | -0.080719351 |
| Middle temporal | 0.883991973 | 0.809581633 | 0.097162558 | -0.883991973 | | 0.550776182 | -0.415943968 |
| Parahippocampal | 0.748561424 | **0.000185844**** | -0.385967011 | -0.883991973 | | 0.416503933 | -0.486449987 |
| Superior temporal | -0.734015778 | 0.710864873 | -0.228907216 | -0.913986433 | | 0.077337416 | -0.179183607 |
| Temporal pole | -0.100190265 | -0.664734323 | **-0.037008971** | -0.824087262 | | -0.826484053 | -0.240761461 |
| Transverse temporal | 0.89315029 | -0.427108862 | **0.005264502** | 0.742534181 | | 0.747033991 | 0.405896424 |
| Insula | -0.883991973 | -0.318046629 | 0.161369756 | -0.863501687 | | -0.305887228 | 0.54825444 |
| *OCCIPITAL* |  |  |  |  | |  |  |
| Cuneus | -0.824087262 | -0.346573292 | 0.12113851 | -0.734015778 | | -0.586496392 | 0.260915084 |
| Lateral occipital | 0.283184679 | 0.950835015 | **0.00000315**** | 0.870370203 | | -0.557267754 | **0.035997352** |
| Lingual | -0.088428764 | -0.08220607 | -0.413762343 | -0.100190265 | | -0.137321205 | **-0.012092385** |
| Pericalcarine | -0.258776267 | **-0.037279232** | -0.101604111 | -0.162657034 | | -0.142005585 | **-0.00894696** |

^a^34 brain regions were parcellated using FreeSurfer software. Children with *SOS1* mutation were compared to TD controls, values represent FDR-corrected *p*-values and sign of *p*-values represent direction of differences (negative: NS < TD; positive: NS > TD); Bolded value with no asterisk has a *p*-value of <0.05, a bolded value with * has a *p*-value of <0.005, and finally, a bolded value with ** has a *p*-value of <0.001.

^b^Banks of the superior temporal sulcus

**References**

1 Marshall WA, Tanner JM. Variations in the pattern of pubertal changes in boys. *Arch Dis Child* 1970; **45**: 13–23.

2 Kaufman J, Birmaher B, Brent D, Rao U, Flynn C, Moreci P *et al.* Schedule for affective disorders and schizophrenia for school-age children-present and lifetime version (K-SADS-PL): Initial reliability and validity data. *J Am Acad Child Adolesc Psychiatry* 1997; **36**: 980–988.

3 Rosen AFG, Roalf DR, Ruparel K, Blake J, Seelaus K, Villa LP *et al.* Quantitative assessment of structural image quality. *Neuroimage* 2018; **169**: 407–418.
